# Pressure ulcers microbiota dynamics and wound evolution

**DOI:** 10.1038/s41598-021-98073-x

**Published:** 2021-09-16

**Authors:** Catherine Dunyach-Remy, Florian Salipante, Jean-Philippe Lavigne, Maxime Brunaud, Christophe Demattei, Alex Yahiaoui-Martinez, Sophie Bastide, Claire Palayer, Albert Sotto, Anthony Gélis

**Affiliations:** 1 Virulence Bactérienne et Infections Chroniques, INSERM U1047, Université de Montpellier, Department of Microbiology and Hospital Hygiene, CHU Nîmes, Univ Montpellier, Nîmes, France; 2 Department of Biostatistics, Epidemiology, Public Health and Innovation in Methodology (BESPIM), CHU Nîmes, Univ Montpellier, Nîmes, France; 3 Centre Mutualiste Neurologique Propara, 34090 Montpellier, France; 4 Department of Infectious and Tropical Disease, CHU Nîmes, Univ Montpellier, Nîmes, France; 5 Department of Orthopedic and Traumatic Surgery, CHU Nîmes, Univ Montpellier, Nîmes, France

**Keywords:** Clinical microbiology, Microbial communities, Infection, Bacterial infection

## Abstract

Bacterial species and their role in delaying the healing of pressure ulcers (PU) in spinal cord injury (SCI) patients have not been well described. This pilot study aimed to characterise the evolution of the cutaneous microbiota of PU in SCI cohort. Twenty-four patients with SCI from a French neurological rehabilitation centre were prospectively included. PU tissue biopsies were performed at baseline (D0) and 28 days (D28) and analysed using 16S rRNA gene-based sequencing analysis of the V3–V4 region. At D0, if the overall relative abundance of genus highlighted a large proportion of *Staphylococcus, Anaerococcus* and *Finegoldia* had a significantly higher relative abundance in wounds that stagnated or worsened in comparison with those improved at D28 (3.74% vs 0.05%; p = 0.015 and 11.02% versus 0.16%; p = 0.023, respectively). At D28, *Proteus* and *Morganella* genera were only present in stagnated or worsened wounds with respectively 0.02% (p = 0.003) and 0.01% (p = 0.02). Moreover, *Proteus, Morganella, Anaerococcus* and *Peptoniphilus* were associated within the same cluster, co-isolated from biopsies that had a poor evolution. This pathogroup could be a marker of wound degradation and *Proteus* could represent a promising target in PU management.

## Introduction

Pressure ulcers (PU) are defined as localised damage to the skin and/or underlying tissue resulting from long-term pressure, or pressure in combination with shear or friction^1^. PUs are a public health problem, affecting approximately 20% of long-term care patients^2^. Prevention or treatment of these wounds generates high healthcare costs^3^. PUs particularly affect the older population, those with neurological impairment (especially spinal cord injury (SCI)), and patients in intensive care units and palliative care^4,5^. In patients with SCI, the presence of a PU can quadruple healthcare costs and increase hospitalisation duration six fold^6^. The management of PU is multidisciplinary. Treatment depends on the patient's state of health and the clinical presentation of the chronic wound. Indeed, PUs vary according to both external (e.g. pressure, friction, moisture) and internal factors (e.g., malnutrition, anaemia, endothelial dysfunction)^7,8^. Moreover, restriction of blood flow due to mechanical compression of surrounding cutaneous tissue unbalances the patient’s immunological response causing inflammation and inhibiting wound healing^9^. Whilst prevention is the key factor in the management of PU, effective management of the wound to limit a delay in healing is also essential^10^. Despite significant progress, some of the mechanisms compromising ability to heal remain unclear, including bacterial colonisation^11,12^. Indeed, chronic wounds are naturally colonised by commensal bacteria from the cutaneous microbiota, with high polymicrobism^13–17^. The main objective for clinicians is to distinguish colonisation from infection^18,19^. Wound microbiota is known to play a significant role in the protection against infection, acting as a "shield" against the implantation of pathogenic bacteria^20^. However, this beneficial impact is questioned. Colonising and pathogenic bacteria present in PUs are organised into Functionally Equivalent Pathogroups (FEP)^15^. These FEPs involve close interaction between pathogenic and commensal bacteria, and consequently some that would be considered incapable of maintaining a chronic infection, could co-aggregate symbiotically in a “pathogenic biofilm” and act synergistically to cause a chronic status^21–23^ and delay healing^15^. Characterising the bacteria participating in a FEP and understanding their roles are important goals in the better management of chronic wounds.

Recently, advanced sequencing technologies have allowed extensive description of the cutaneous microbiota diversity. A metabarcoding approach targeting universal 16S rRNA sequences has been applied to describe some FEPs, particularly in diabetic foot ulcer and venous leg ulcer^15,24,25^. Smith et al*.* identified 487 bacterial taxa and demonstrated the importance of *Corynebacteria* and strict anaerobic bacteria in PUs. Moreover, the authors observed no correlation between clinical data and bacterial clusters^26^. Few data are available on the characterisation of the PU microbiota^26,27^ and the monitoring of wound evolution.

The aim of this study was to monitor the wound microbiota over time and evaluate its influence on the evolution of PU in SCI patients using a metagenomics approach.

## Results

### Studied population

In total, 55 patients with SCI and PU were prospectively included between May 2015 to December 2017. One patient was withdrawn from the analysis, requiring surgical management between D0 and D28 and one was lost-to-follow-up. Metagenomic analysis was of insufficient quality for accurate interpretation for the criteria examined in this study for 29 patients, thus only the 24 with high-quality data were retained (Table S1). Twenty-four patients with pelvis PU were therefore analysed (15 males, 9 females; median age: 61.5 (31–89) years) (Table 1). Localisation of PU was ischiatic (n = 12), sacral (n = 11) and trochanteric (n = 1). At D28, 15 (62.5%) patients showed improved PU and 9 (37.5%) presented a stagnated or worsened PU. No difference between groups was noted regarding antibiotic intake between D0 and D28, nutritional status (Body Mass Index, weight change, undernutrition score), age and wound characteristics (wound stage, localisation, number of PU, PU area and depth) (Table 1).

**Table 1:** Clinical data of included patients in total population and according to wound evolution (improved vs stagnated/worsened).

Variable		Total population (n = 24)	Improved (n = 15)	Stagnated/worsened (n = 9)	P-value
Sex	Female	9 (37.5%)	8 (53.3%)	1 (11.1%)	0.0803
Age	Years	61.5 (55.5, 67)	64 (57.5, 67.5)	54 (49, 66)	0.148
Body mass index (kg/m^2^)	< 18.5	2 (8.3%)	1 (6.7%)	1 (11.1%)	0.933
	18.5–25 <	7 (29.2%)	4 (26.7%)	3 (33.3%)	
	25–30 <	10 (41.7%)	7 (46.7%)	3 (33.3%)	
	≥ 30	5 (20.8%)	3 (20%)	2 (22.2%)	
Weight change (%)	% reduction^a^	0 (− 1.3, 1.1)	0 (− 1.01, 1.70)	0 (− 1.2, 0)	0.346
Undernutrition	None	9 (37.5%)	5 (33.3%)	4 (44.4%)	0.649
Score^b^					
	Moderate	6 (25%)	5 (33.3%)	1 (11.1%)	
	Severe	9 (37.5%)	5 (33.3%)	4 (44.4%)	
CRP (D0)	mg/l	16.5 (8.5, 40.75)	22 (9, 46.5)	13 (9,22)	0.633
CRP (D28)		14 (5.5, 23.5)	20 (5.5, 23.5)	11 (6.25, 23)	0.747
Wound stage (D0)	III	16 (66.7%)	9 (60%)	7 (77.8%)	0.657
	IV	8 (33.3%)	6 (40%)	2 (22.2%)	
Wound stage (D28)	II	2 (8.3%)	2 (13.3%)	0 (0%)	0.678
	III	15 (62.5%)	9 (60%)	6 (66.7%)	
	IV	7 (29.2%)	4 (26.7%)	3 (33.3%)	
Wound localisation	Ischial	12 (50%)	7 (46.7%)	5 (55.6%)	
	Sacral	11 (45.8%)	8 (53.3%)	3 (33.3%)	0.384
	Trochanteric	1 (4.2%)	0 (0%)	1 (11.1%)	
Area of wound^c^	% reduction^a^	− 22.19 (− 45.9, 15.98)	− 40.2 (− 55.46, 23.22)	5.88 (− 19.37, 15)	0.138
Depth of wound	% reduction^a^	− 17.78 (− 55, 40)	− 40.59 (− 57.5, − 4.44)	33.33 (− 2.22, 50)	0,185
Duration of wound	Months	6.26 (2.52, 16.21)	6.28 (3.42, 14.89)	6.02 (1.77 14.63)	0.698
Detachment (D0)	Yes (n; %)	13 (54.2%)	10 (66.7%)	3 (33.3%)	0.206
Detachment (D28)	Yes (n; %)	13 (54.2%)	9 (60%)	4 (44.4%)	0.675
Amount of exudate (D0)	None	0 (0%)	0 (0%)	0 (0%)	0.334
	+	9 (37.5%)	4 (26.7%)	5 (55.6%)	
	++	9 (37.5%)	6 (40%)	3 (33.3%)	
	+++	6 (25%)	5 (33.3%)	1 (11.1%)	
Initial treatment	Yes (n; %)	12 (50%)	9 (60%)	3 (33.3%)	0.4
Antibiotherapy	Yes (n; %)	5 (20.8%)	4 (26.7%)	1 (11.1%)	0.615

Quantitative variables are expressed as median (Q1, Q3) and qualitative variables as absolute frequency (relative frequency in %). The statistical tests are either Student or Wilcoxon for quantitative variables and Chi-squared or Fisher’s exact for qualitative variables.

^a^100 × (D28-D0)/D0, ^b^in accordance to^28^, ^c^Kundin’s formula^29^.

### Description of bacterial microbiota isolated from wounds at the enrolment

A total of 2,177,113 paired-end reads were generated. The percentage of 16S rDNA reads clustering at 97% of identity was 77% (Table S1). 1,763,282 sequences were conserved for clustering into 1277 (Operational Taxonomic Unit) (OTUs) (685 at D0 and 592 at D28) (Table S2). For diversity index calculation, the number of sequences from which the diversity of the sample was exhaustive was evaluated at 11,422 sequences. Sequences have been deposited on NCBI (Table S3).

**Figure 1:**
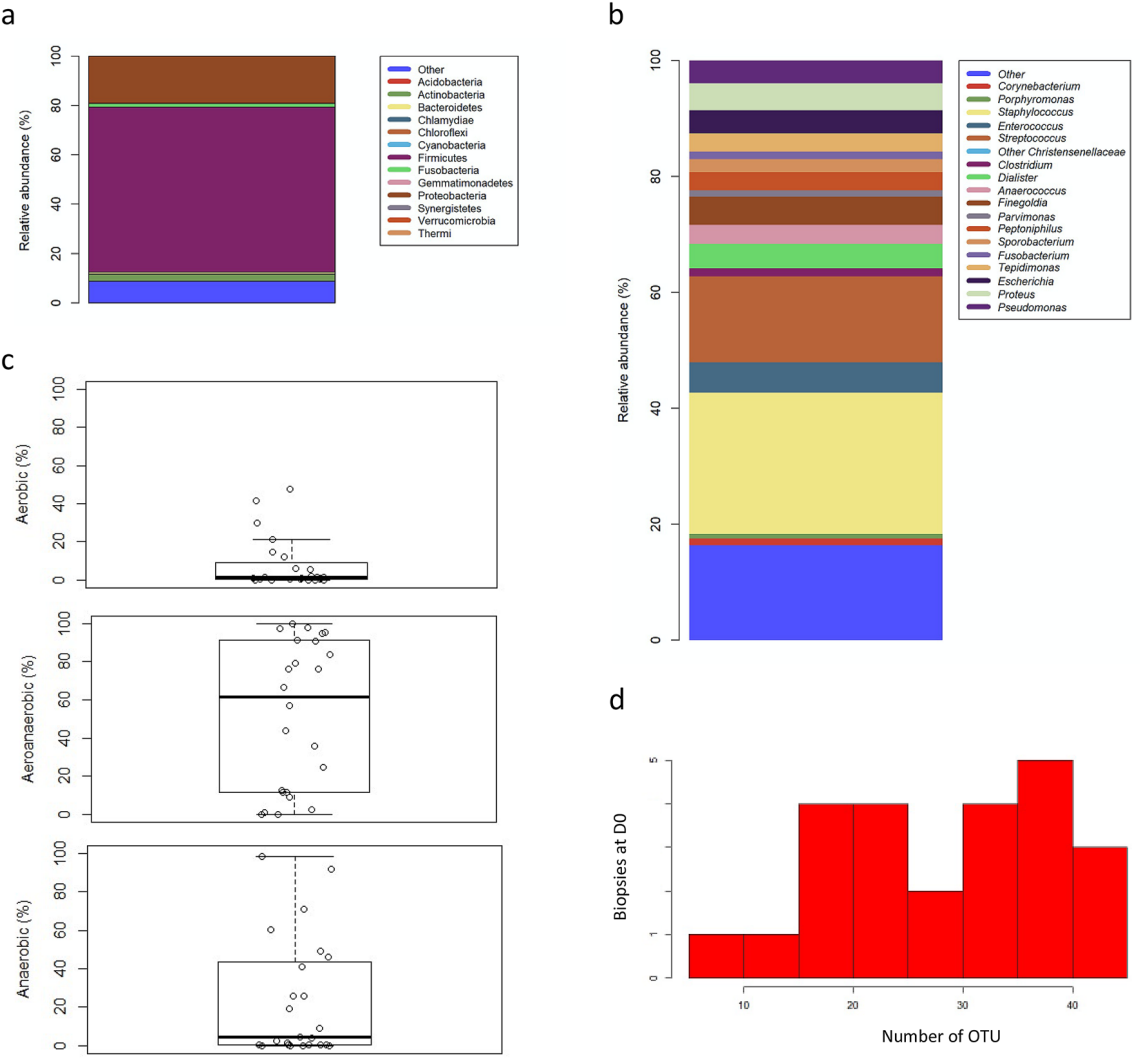
Description of bacterial communities isolated from wounds at D0. (**a**) Barplot of overall repartition of phyla in wounds at D0 for total population (n = 24). (**b**) Barplot of average relative frequencies of genera. Only genera > 1% of OTUs in at least one wound and in at least one time (D0 or/and D28) were represented. The remaining genera are added to the group “other”. (**c**) Boxplot of genus relative frequencies gathered in respiratory phenotype. (**d**) Histogram of OTU’s number by wounds at D0**.**

The repartition of phyla in PU at enrolment (D0) showed a large proportion of *Firmicutes* (80.47% (44.17–94.76)), followed by *Proteobacteria* (6.54% (0.71–49.67)) and *Actinobacteria* (0.98% (0.11–3.28)) (Fig. 1a). The average relative abundance of genera highlighted a large proportion of *Staphylococcus* (24.38%), followed by *Streptococcus* (14.86%), *Enterococcus* (5.2%), *Finegoldia* (4.83%), *Dialister* (4.17%) and *Anaerococcus* (3.3%); all these genera belonging to *Firmicutes* phyla (Fig. 1b). Concerning *Proteobacteria*, *Proteus* was the genus most frequently isolated in the PU (4.66%) followed by *Escherichia* (3.96%) and *Pseudomonas* (3.96%). Finally, among *Actinobacteria*, *Corynebacterium* was the most prevalent genus with an average relative abundance of 1.17%.

Analysis of the oxygen tolerance phenotype of bacteria present in PU at D0 showed that the median percentage of facultative anaerobic and aerobic bacteria was 61.73% (11.71; 91.03) and 1.1% (0.130; 7.362), respectively (Fig. 1c). While the average percentage of strict anaerobic bacteria was 23%, the median was lower (4.18% (0.265; 42.15)). We observed a high individual variability in this percentage, with some biopsies presenting a high rate of strict anaerobic bacteria (reaching 98.64%) and others a complete absence (Fig. 1c). Finally, the median number of OTU per wound was 31.5 (20.75; 37) (Fig. 1d).

### Bacterial microbiota, undernutrition and wounds characteristics

The wound microbiota was analysed depending on wound characteristics (duration, depth, area, stage, localisation) and the presence/absence of undernutrition at enrolment (Table S4). Overall, no change was observed in bacterial composition and diversity according to wound characteristics and patient nutritional status. Some bacterial genera were more present in deep wounds (> 15 mm) such as *Dietzia* (p = 0.04), *Paracoccus* (p = 0.04), *Nosophingobium* (p = 0.003), *Delftia* (p = 0.0158) and *Pelomomas* (p = 0.004) or in large wounds (> 600 mm) such as *Brevibacterium* (p = 0.03) (Table S4). Concerning bacterial diversity, Shannon and Chao’s indexes and the OTU number did not significantly vary irrespective of the wound characteristics except for wound depth. Indeed, for wounds deeper than 15 mm, the Chao index and the number of OTU were significantly higher, showing greater bacterial diversity in deep wounds versus superficial wounds (Chao index = 96 (69, 175.25) vs 40 (33, 79) (p = 0.03; q = 0.05, AUC = 0.77); number of OTU = 34.5 (28, 37.25) vs 21 (17, 30) (p = 0.02; q = 0.05, AUC = 0.77) respectively).

### Evolution of bacterial microbiota after 28 days

The evolution of wound microbiota was studied between D0 and D28. No change in bacterial diversity was noted between these two periods. A subgroup analysis was performed on the patients treated by antibiotics (n = 5). The small group size limits the statistical power, preventing drawing of strong conclusions, but it appears that no clear evidence for change in diversity and composition of the microbiota is observed: 150 (73, 153) at D0 vs 138 (40, 146) at D28 (Table S5).

**Figure 2:**
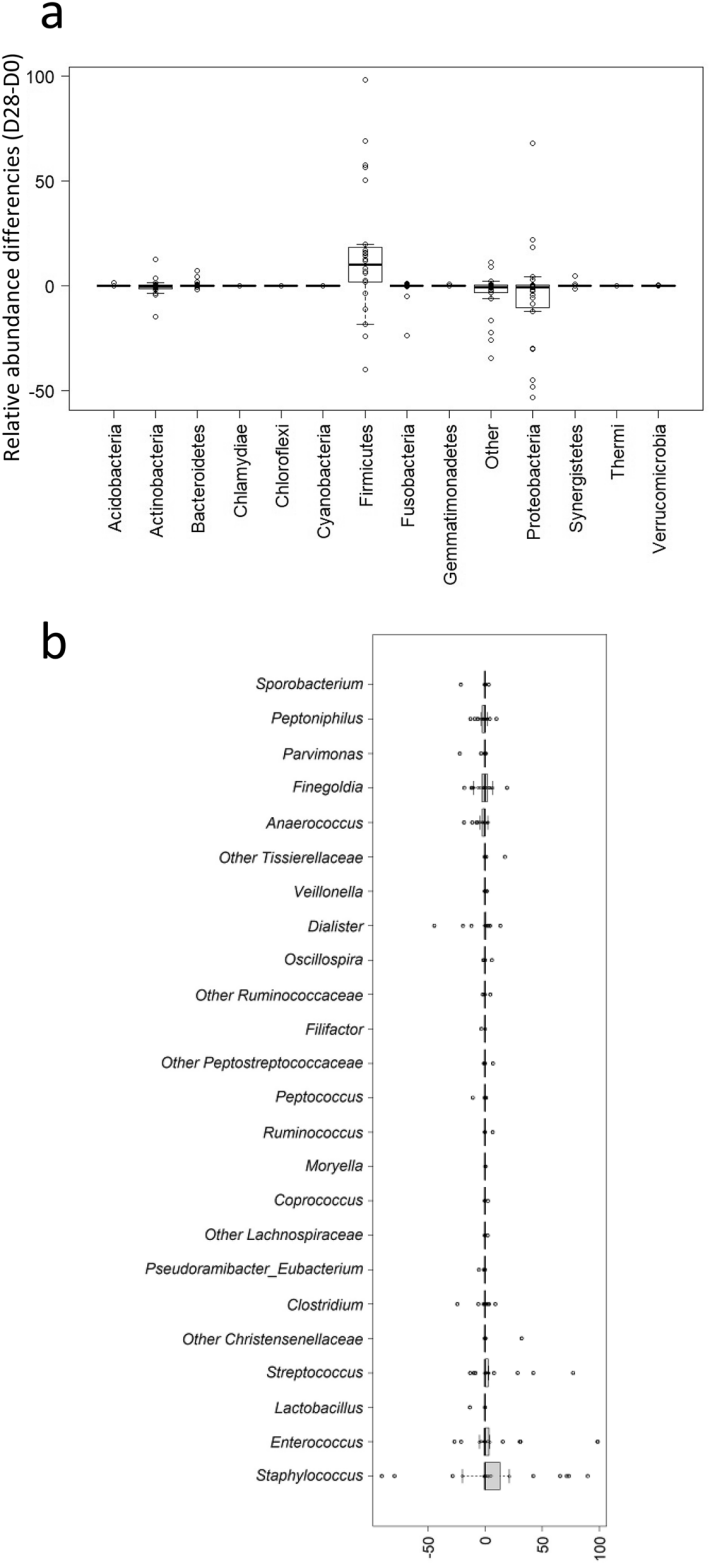
Evolution of bacterial microbiota isolated from wounds over time. (**a**) Boxplot of relative frequencies differences (D28-D0) of phyla (the group “Other” represents unclassified bacteria at phylum level). (**b**) Boxplot of relative frequencies differences (D28-D0) of bacteria genera. Only genera > 1% of OTUs in at least one wound and in at least one time (D0 or/and D28) and belonging to Firmicutes are represented.

Interestingly, the study of phyla showed that the number of bacteria belonging to the *Firmicutes* increased significantly over time (74.9% (42, 89) at D0 vs 92.9% (65.6, 99.2) at D28, (p = 0.0164)) (Fig. 2a). We notably observed an increase of the genera *Staphylococcus, Streptococcus* and *Enterococcus* between the two periods, although this increase did not reach statistical significance (Fig. 2b, Table S5). However, we also noted that the genus *Anaerococcus* was significantly reduced at D28 (p = 0.04). The same trend was noted for *Peptoniphilus* but without significance (p = 0.24; AUC = 0.57) (Fig. 2b, Table S5). A decrease of the *Proteobacteria* phyla was observed between the two periods, but again failed to reach significance (5.45% (0.6, 41.1) vs 2.5% (0.1, 13.1), p = 0.10). However, two genera decreased significantly over time: *Sphingomonas* (p = 0.0215; AUC = 0.64) and *Delftia* (p = 0.045, AUC = 0.61) (Table S5).

### Evolution of microbiota according to the clinical evolution of the wound

Patients were classified into two groups according to clinical evolution of wounds: 'Improved' (n = 15) and 'Stagnated/Worsened’ (n = 9) at D28. No difference was observed in the evolution of the diversity of the wound microbiota (Shannon or Chao indexes and number of OTU) according to the clinical evolution of the wound (Table S5). The beta diversity showed that a significantly different microbiome was observed according to patient's wound evolution (p = 0.039) (Fig. S1). No significant evolution of the microbiomes of patients were noted between D0 and D28 (p = 0.136). However, we observed a trend for the interaction between wounds evolution and time but at the limit of significance (p = 0.059). The microbiomes were different between patients with improved, stagnated or worsened wounds but no statistical evolution could be noted between D0 and D28.

**Figure 3:**
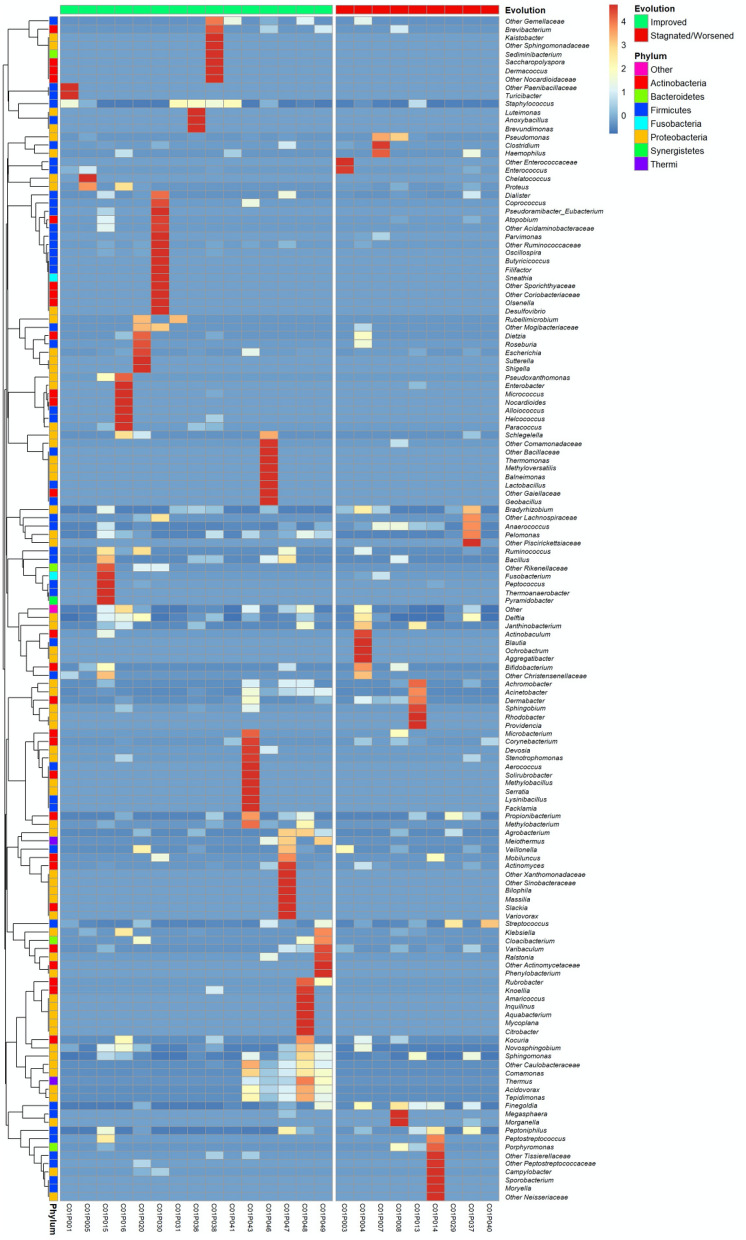
Heatmap of the PU microbiota’s standardised composition at D0 ranked according to wound evolution. Standardised relative frequencies of genera are used in order to see variations between groups even for low-abundant bacteria. Genera are classified according to agglomerative hierarchical clustering with complete linkage.

The relative abundance of two strict anaerobic bacteria, *Anaerococcus* and *Finegoldia*, was significantly higher at D0 in wounds with a “Stagnated/Worsened” evolution: 3.74% (1.04; 11.04) vs 0.05% (0.005; 0.7); (p = 0.015; q = 0.66) and 11.02% (1.27; 13.41) versus 0.16% (0.01; 1.29); (p = 0.023; q = 0.78), respectively (Fig. S2a,b). Moreover, *Peptoniphilus* and *Streptococcus* also had a higher relative abundance at D0 in wounds with a “Stagnated/Worsened” evolution, but without significant results: 1.19% (1.05; 6.6) vs 0.03% (0; 1.17); (p = 0.1; AUC = 0.7) and 0.65% (0.05; 13) vs 0.14% (0.05; 9.21); (p = 0.38; AUC = 0.61) respectively. However, these four genera did not belong to the same cluster, and thus were not isolated simultaneously from the same wound (Fig. 3). Their presence was independent of each other. No significant difference could be observed in the distribution of strict anaerobic, facultative anaerobic and aerobic bacteria according to the clinical evolution of the wound. However, the percentage of strict anaerobic bacteria at D0 was higher in wounds with “Stagnated/Worsened” evolution compared to wounds with “Improved” evolution (25.7% (4.4, 45.9) versus 1.6% (0.24, 14.05), p = 0.144, AUC = 0.685, respectively). In contrast, the percentage of facultative anaerobic bacteria at D0 was higher in wounds with “Improved” evolution in comparison with wounds with “Stagnated/Worsened” evolution: 76.3%, (24.2, 93.1) vs 24.6% (11.6, 83.6), p = 0.47, AUC = 0.59 respectively.

Microbiota composition at D28 according to the clinical evolution of the wounds showed that the genus *Pelomonas* was only detected in wounds with “Improved” evolution (0.01% (0; 0.03); p = 0.04, AUC = 0.7). Although not reaching statistical significance, a difference was observed in abundance of *Corynebacterium* in wounds that improved than in those with “Stagnated/Worsened” evolution: 0.13% (0.015, 0.39) vs 0.01% (0, 0.007); p = 0.194, AUC = 0.66. In contrast, *Proteus* and *Morganella* genera were only present in wounds that had a “Stagnated/Worsened” evolution with respectively 0.02% (0, 0.85, p = 0.003, AUC = 0.8) and 0.01% (0; 0.03, p = 0.02, AUC = 0.67) (Fig. S2c,d). A higher proportion of *Anaerococcus* and *Peptoniphilus* was also noted in these wounds but without significant results: 0.64% (0.06, 3.18) vs 0.01% (0.005, 0.35) (p = 0.15, AUC = 0.68) and 0.12% (0.03, 5.31) vs 0.06% (0, 0.45) (p = 0.15; AUC = 0.68), respectively.

**Figure 4:**
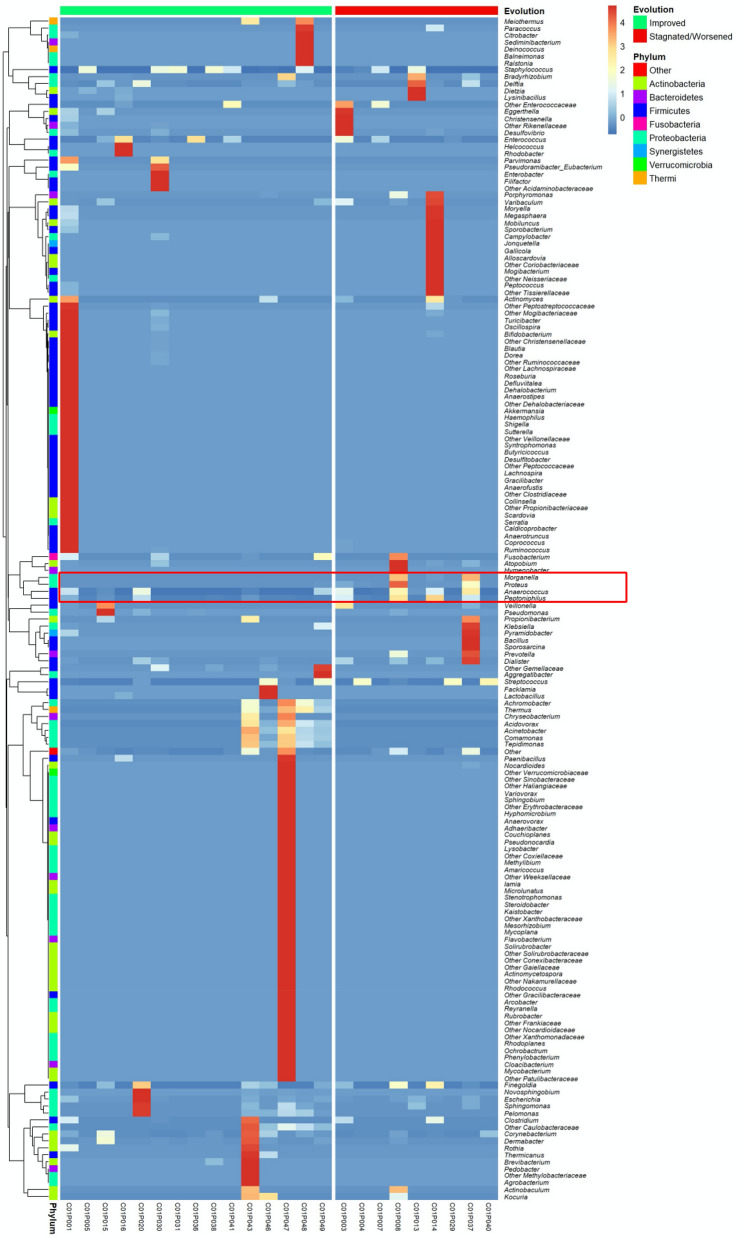
Heatmap of the PU microbiota’s standardised composition at D28 ranked according to wound evolution. Standardised relative frequencies of genus are used to see variations between groups even for low-abundant bacteria. Genera are classified according to agglomerative hierarchical clustering with complete linkage.

**Figure 5:**
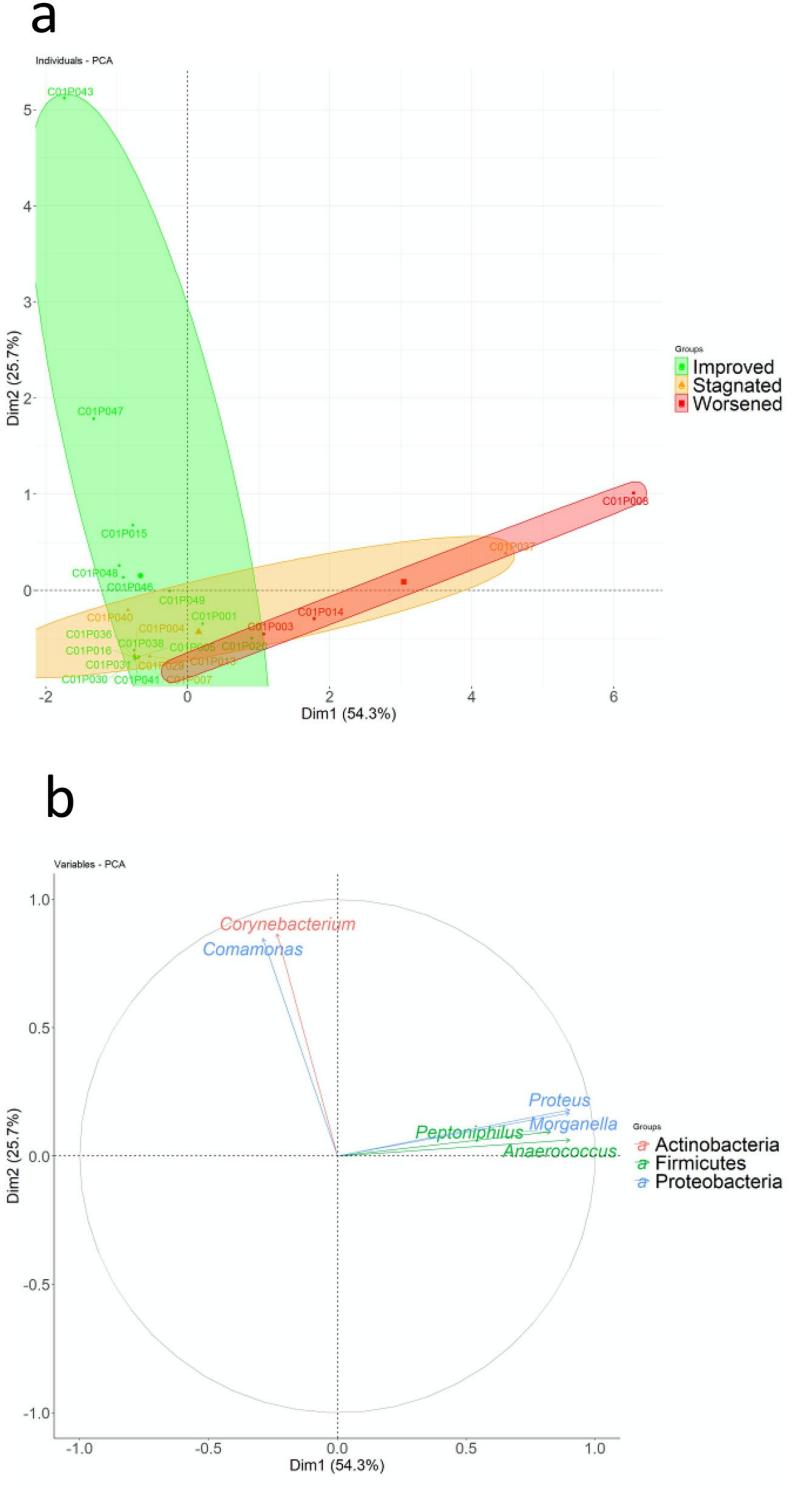
Principal Component Analyses (PCA) of the most discriminant genus. The principal component analysis is based on standardised data for a selection of genera with the best ability to separate wounds according to their evolution (“Improved” “Stagnated” and “Worsened”). The criterion for the genus selection is an Area Under the ROC Curve greater than 0.65. The two first components are shown, representing 80% of total information.

*Proteus*, *Morganella*, *Anaerococcus* and *Peptoniphilus* were associated within the same cluster (Fig. 4). These four genera were frequently co-isolated in biopsies with “Stagnated/Worsened” evolution. In PCA representation, which the two main components represent 80% of the total information, we noted that the wounds with Stagnated/Worsened evolution were linked to the presence of *Proteus, Morganella Anaerococcus* and *Peptoniphilus* cluster, whereas the wounds with Improved evolution were linked to the presence of the genera *Comamonas* and *Corynebacterium* (Fig. 5). However, these two last genera did not belong to the same cluster (Fig. 4). Finally, neither the use of alginate dressings, nor the use of antibiotics influenced the bacterial diversity and wound microbiota composition at D28 (Table S6).

### Evolution of bacterial genera and wound over time

We showed that *Proteus* decreased significantly between D0 and D28 in wounds with “Improved” evolution (0.1 (0, 0.165) vs 0 (0, 0); p = 0.036), and was absent at D28 (Fig. S3a), whereas this genus remained relatively constant in “Stagnated/Worsened” wounds over time (0.66 (0, 0.76) vs 0.02 (0, 0.85); p = 0.8) (Fig. S3a). *Corynebacterium* decreased significantly over time in wounds with “Stagnated/Worsened” evolution ((0.23% (0.02, 1.82) at D0 versus 0.01% (0, 0.07)) at D28, (p = 0.035)), whereas this bacterial genus increased not significantly in wounds with “Improved” evolution ((0.02% (0, 0.12) at D0 vs (0.13% (0.015, 0.35)) at D28 (p = 0.328) (Fig. S3b). Finally, the percentage of aerobic bacteria decreased in “Stagnated/Worsened” wounds (1.03% (0.13; 5.53) versus 0.09% (0.02, 0.95), p = 0.073), the p value being at the limit of significance.

## Discussion

Commensal microbiota has been shown to play a crucial role in healing of chronic wounds. These bacteria help to maintain a balance within the bacterial communities present in the wound by limiting implantation or modulating the virulence of known pathogens^30,31^. However, in this dynamic microbial community, a complex competitive or synergistic interaction between commensal and pathogenic microorganisms plays a major role on the severity and the evolution of the wound^32^. Some bacterial communities are implanted more permanently than others within wounds and evolve over time^33^.

At baseline, the main characteristics of PU microbiota described in our pilot study were similar to those observed previously, with a great diversity of microbiota between wounds (Fig. 1d) and the predominance of eight genera (*Streptococcus, Corynebacterium, Staphylococcus, Finegoldia, Anaerococcus, Pseudomonas* and *Peptoniphilus*), of which the majority belonged to *Firmicutes* (Fig. 1a)^16,27^*.* The main difference was the lower percentage of *Pseudomonas* and *Corynebacterium* observed in our study compared to the others (3.96% vs 19% and 1.17% vs 11%, respectively)^27^. This difference could be explained by the sampling method used. In our study, all deep tissue biopsies were performed after debridement, whereas tissue debridement was studied in the other previous works. This physical treatment of the infected tissue removes a large part of commensal bacteria (including *Corynebacteria*) but also the transient bacteria such as *Pseudomonas,* an aerobic species mainly present at the surface of the wounds^34^. Other important results were the presence and the relative abundances of strict anaerobic bacteria in wound microbiota. The development of molecular tools has demonstrated that conventional bacterial cultures underestimated these species^35,36^. In our study, their relative abundances were similar to those observed by Smith et al*.* (23% versus 31%)^26^, but lower than those described by Dowd et al*.* (~ 60%)^16^. We also noted a large variation in abundances between individuals, varying between 0 to 98%. This result suggests that there is not a single type of bacterial community in PU, but rather different communities with variable proportions of anaerobic bacteria^33^.

One of the main findings of our study was the link between the presence/absence of bacterial genus and the worsened/improved evolution of the wounds. Firstly, two strict anaerobic genera, *Finegoldia* and *Anaerococcus*, were significantly present in wounds with a worsened evolution, despite not belonging to the same cluster (Figs. 3 and S1). This suggests that these two genera could affect wound prognosis, as previously noted^25^. In contrast, the facultative anaerobic genus *Streptococcus* was associated with wounds with worsened evolution after 28 days (Fig. 3) but not significantly (p = 0.38). McDonald et al*.* have previously observed that high abundance of strict anaerobic genus (belonging to the *Bacteroidales* family) and *Streptococcus* was significantly associated with lack of wound healing^37^. However, this result was not observed in other studies^25,38^. Secondly, our study suggests that the initial composition of microbiota could help predict the evolution of the wounds. The genus *Corynebacterium* was positively correlated with wounds with an improved evolution at D28 and decreased in wounds with delayed healing (Fig. S2). *Corynebacteria* belong to commensal microbiota and have been show to modulate the virulence of pathogenic bacteria such as *Staphylococcus aureus*^20^. Our data confirmed the important role of the commensal bacteria, notably in the maintenance of the equilibrium of the wound microbiota. Thirdly, four bacterial genera (*Proteus, Morganella, Anaerococcus* and *Peptoniphilus*) were associated with wounds with a worsened evolution (Fig. 4). Moreover, we observed that these bacterial genera were associated together in a cluster constituting a FEP (Fig. 5). Dowd et al*.* described eight bacterial clusters in their samples, including one comprising a facultative anaerobic genus *Serratia* associated with strict anaerobic genera (*Finegoldia, Peptoniphilus and Anaerococcus*)^15^. As *Serratia* belongs to Enterobacteriales such as *Proteus* and *Morganella,* we suggest that this group of bacteria possesses a high potential to participate in FEPs and to favour non-healing sores. Among FEPs, strict anaerobic bacteria were the most frequently found species. Dowd et al*.* previously highlighted that these bacteria were detected in 7 out of 8 FEPs^15^. Strict anaerobic bacteria have been described to co-aggregate with aerobic or facultative anaerobic bacteria in symbiotic associations where the aerobic or facultative anaerobic bacteria consuming oxygen created a localised niche improved to the development and/or persistence of strict anaerobic bacteria^39^. This symbiotic relationship could partially explain why the FEP found in our study comprised the enterobacteria *Proteus*, *Morganella* and the strict anaerobic genera. Inside the FEPs, bacteria are organised in a biofilm, a status frequently observed in chronic wounds (60–80%)^23,40^. Bacteria within the biofilm evade the host’s natural defences and are resistant to antibiotics and host immune defence^40^. Strict anaerobic bacteria establish synergistic relationships to obtain nutrients, or adapt to the environment^41^. Two bacteria, *Anaerococcus* and *Peptoniphilus* have the potential to metabolise peptones and amino acids and to produce short-chain fatty acids (SCFAs) such as butyric acid. These bacteria interact with polynuclear neutrophils (PNN) which act as the primary host defence against pathogens. Certain strains of *Bacteroides* are known to decrease the phagocytosis of PNNs through butyric acid production, and thus *Proteus mirabilis* are less phagocytosed by these cells^42^. Indeed, SCFAs alter the migration and chemotactic mobility of PNNs and inhibit the response of PNNs by altering degranulation and the production of oxygenated free radicals. These SCFAs also alter the membrane surface of PNNs, which emits cytoplasmic projections^42^. Thus, SCFAs could play a central role in the interaction between strict anaerobic bacteria and their microenvironment and in the virulence among polymicrobial infections. Extrapolating from the interaction between *Bacteroides* and *Proteus* and the consequence on the functions of PNNs, we could hypothesize a similar collaboration between the pathogroup constituted by *Anaerococcus, Peptoniphilus, Proteus* and *Morganella,* and identified in our study. We also observed that the high abundance of *Proteus* was not directly correlated with wounds with a worsened evolution, but rather with their persistence within the wound. We observed that wounds with a worsened evolution had an initially high percentage of *Anaerococcus.* We could hypothesise that this genus creates an enabling environment for the implantation and survival of *Proteus,* and butyric acid could help maintain *Proteus* and *Morganella*. Further investigations must confirm this trend.

Although these results provide a better understanding of the impact of bacterial interactions in wound evolution, several limitations must be taken into account. First, this work was a pilot study with a limited number of included patients (n = 55) and analysed (n = 24). A significant number of samples of patients were not included because the sequencing was low quality. This was due to a low number of raw sequences obtained or to an initial high human DNA contamination. On the other hand, in this study, the *q*-values (FDR adjusted *p*-values) of the tests have been reported by convention but only *p*-values have been considered. Indeed, given the sample size and the underlying statistical power, and the high tests number (250 bacteria), it is very unlikely to have a q-value < 0.05. Only p-values < 10^–4^ may appear statistically significant after adjustment. In this context, the AUCs have been reported in order to give an additional information about the discriminatory power of the variables. A study with a larger number of patients will have to be conducted to confirm these initial results. To highlight a difference between D0 and D28 in change of *Proteus* abundance of 7.74 between improved and stagnated patients with a standard deviation of 13.00, an alpha risk of 5%, a power of 90%, and a ratio of worsened to stagnated of 0.6, 130 patients should be require (81 improved and 49 stagnated patients), without take account the rate of non-analysable sequences with potential poor-quality.

However, this study opens new avenues for diagnosis and management of chronic wounds. Routinely performing molecular tests would offer clinicians personalised diagnosis and treatment. Thus, the presence of certain types of strict anaerobic bacteria (such as *Finegoldia* and *Anaerococcus*) associated with enterobacteria characterising poor wound evolution, would trigger specific management with a sharp and repeated debridement to prevent the formation of FEPs. Our results also showed the low impact of oral antibiotic therapy on the diversity and composition of the microbiota (Table S5), as previously described by Smith et al.^26^. Antibiotic therapy, in the absence of clear signs of infection, has no benefit on restoring a balance within the bacterial communities in the wounds. Personalised wound management represents the future direction to improve and decrease the delay of healing. It could also reduce wound-related costs and resource use, improve quality of life and patient outcomes. Personalised management would help improve antibiotic stewardship and represents an interesting strategy to combat antibiotic resistance.

## Conclusion

Although a multidisciplinary approach is essential for the successful management of PU, this pilot study highlighted the central role that wound microbiota could play in wound healing, notably the presence or absence of strict anaerobic bacteria. Due to the difficulty in cultivating these species, the development of molecular tests will be pivotal in improving the management of chronic wounds. These data also confirmed that the study of bacterial interactions within the wound is essential to understand their pathophysiology and could spur development of innovative diagnostic tools and treatments.

## Methods

### Patients and samples

The present study was approved by the local ethics committee (South Mediterranean III; N°2014.04.01bis January 25, 2017). Patients provided written informed consent upon enrolment. This trial was registered April 2, 2014 at clinicaltrials.gov NCT0205. All experiments were performed in accordance with relevant guidelines and regulations.

This work was an observational, prospective and monocentric pilot study. Patients aged 18–65 years with SCI and a grade NPUAP (US National Pressure Ulcer Advisory panel) 3 or 4 PU, with no prior antibiotherapy within the 2 weeks before enrolment were eligible for the study. Patients were recruited by a single investigator (AG) in the Centre Mutualiste Neurologique Propara (Montpellier, France) after written consent. Only patients requiring modern wound dressings could be included (i.e. not tulle or gauze), without bactericidal or antibiotic elements. Patients with a grade 1 or 2 PU were excluded, as were those receiving anticoagulation therapy. Patients could only be included in the study once and only the PU with the highest stage was considered.

Clinical data and wound samples were collected at enrolment (day (D)0) and at study end (day (D) 28). Clinical data included neurological assessment, nutritional status (Body Mass Index and serum albumin level) and wound assessment. Nutritional status also included the undernutritional score as described by Cederhom et al.^28^. Wound assessment was both qualitative (RYB (Red-Yellow-Black) wound classification^43^) and quantitative (maximal length and maximal perpendicular width, depth using a ruler). These qualitative and quantitative criteria were used to classify wounds into “Improved” and “Stagnated/Worsened” groups. Wound stage and localization were also noted, as was exudate amount and detachment. Any antibiotic use between D0 and D28 and the different types of dressings used were recorded. At D28, the evolution of the wounds was classified into two categories (“Improved” or “Stagnated/Worsened”) on the basis of clinical judgment by the investigator and a specialised nurse in consensus.

Two deep tissue biopsies (3 mm wide and 10 mm deep) were performed by the same investigator (AG), one at D0 and one at D28, using Biopsy punch (3 mm biopsy punch, Kai Europe, Solingen, Germany) after a sharp superficial debridement. The biopsies were then sectioned, placed into DNA Lo-Bind^®^ tubes (1.5 mL) (Eppendorf, Montesson, France) and immediately frozen at − 20 °C. They were transported within a week to the INSERM U1047 and frozen at − 80 °C. Swabs of superficial levels of the bedsores in addition to urine and stool samples were also collected; the results from these analyses will form the basis of a subsequent publication.

### Bacterial DNA extraction

After digestion with proteinase K at 56 °C for 3 h, bacterial DNA was extracted from biopsies obtained at D0 and D28. Tissue samples were lysed using MagNA Lyser Instrument^®^ (Roche, Meylan, France). A 300 µL sample was added into prefilled disposable vials containing ceramic bead compatible with MagNA Lyser and centrifuged twice at 5000 rpm for 60 s. Samples were centrifuged briefly and DNA was extracted from 200 µL of the supernatant using the EZ1 DNA Tissue kit (Qiagen, Courtaboeuf, France) according to the manufacturer’s instructions. DNA was eluted with 100 μL ultrapure Molecular Biology grade water. An extraction control with ultrapure Molecular Biology grade water was used. The concentration of extracted DNA was measured by spectrophotometry (Nanodrop^®^, ThermoScientific, Illkirch, France).

### Bacterial genomes sequencing

The bacterial communities of the gDNA samples were analysed with a metabarcoding approach based on a process developed, optimised and standardised by GenoScreen (Lille, France)^44^. First, amplicon libraries were prepared according to the Metabiote^®^ solution, limiting bias amplifications between samples and including a positive control (bacterial community artificial "ABC control") and a negative control (PCR background noise of the process total library preparation). Extraction controls (PCR-quality water having undergone the same extraction process) were also performed. Libraries were generated targeting the V3-V4 region of the 16S rDNA. The sequencing of the amplicon libraries was performed on a single run Miseq (Illumina^®^, Paris, France) "paired-end" in 2 × 250 base chemistry. After a validation of a quality control of the obtained sequences, demultiplexing was performed by CASAVA (Illumina^®^, Paris, France) software using the PERL script ConfigureBclToFactq.pl. Raw paired-end reads were subjected to the following process: (i) search and removal of both forward and reverse primers using CutAdapt, with no mismatches allowed in the primer sequences; (ii) quality filtering using the PRINSEQ-lite PERL script^45^, by truncating bases with a Phred quality score of < 30; and (iii) paired-end read assembly using FLASH^46^ tool, with a minimum overlap of 30 bases and 97% overlap identity. Following the steps of preprocessing, chimera sequences were detected and eliminated (by an in-house method based on Usearch v6.1).

Similar sequences were clustered at a defined identity threshold (97% identity for genus affiliation on the targeted 16S rDNA region) with Uclust v1.2.22q^47,48^. The database used was Greengenes version 13-8 (http://www.greengenes.gv) by V2.2 method of RDP (Ribosomal Database Project II) classifier^49^. The rarefaction curves highlighted the exhaustive description of the samples when the curve reached the plateau. This identified the number of sequences from which the diversity of the sample was exhaustive. The alpha-diversity was calculated by “Observed OTUs”, “Shannon” and “Chao1” index. These diversity indices represented the taxonomic diversity of samples as the number of sequences obtained increased.

The beta diversity has been addressed with Bray Curtis dissimilarity. A repeated measures PERMANOVA test (adonis function from VEGAN R package) was used in order to evaluate the differences in microbiome according to wound's evolution and time visit while considering the paired structure of the data.

Operational Taxonomic Unit (OTU) table was transformed in relative abundance (%) in function of total sequences per samples. Relative abundances have been considered in order to take into account the total count variability between sample. Therefore, these % values are comparable across samples.

### Statistical analysis

All the statistical analyses were performed with R software (version 4.0.3)^50^. As this is a pilot study, and in the absence of previous data in the literature, a statistical justification for the number of subjects needed was not possible. The initial sample size of 55 patients had to lead to show a difference of 30% between D0 and D28 with a 90% and alpha risk of 5%. Due to the discarded samples, only 24 patients were definitively included in the analyses, this sample size showed a difference of 40% between D0 and D28 with a power of 85% and alpha risk of 5%. For the description of the study population, each variable was described either with median and quartiles (for quantitative variables) or frequency and relative frequency (for qualitative variables). These summaries were presented for each variable in the total study population and in groups according to the evolution of the wound at D28 (Improvement vs Stagnated/Worsened). Variables were compared between groups using Student or Wilcoxon–Mann–Whitney for quantitative variables or Chi-square or Fisher exact for qualitative variables. Statistical significance has been established according to type I error rate of 5%. Bacterial communities’ comparison (D0 vs D28, Improved vs Stagnated/Worsened at D0, Improved vs Stagnated/Worsened at D28, Duration of PU: ≤ 6 months vs > 6 months at D0, Depth of wounds: ≤ 15 mm vs > 15 mm at D0, Area of wounds: ≤ 600 mm^2^ vs > 600 mm^2^ at D0, Wound stage: III vs IV at D0, Malnutrition: No vs moderate/severe at D0, Antibiotherapy: Yes vs No at D28, Dressing: Alginate vs Hydro at D28) was performed using Wilcoxon–Mann–Whitney given the non-normal distribution in most of the cases. The cut-offs for the Depth (15 mm) and Area (600 mm^2^) of wounds were set according to the median (rounded to a meaningful integer value) in order to have groups of equal sizes. For Duration, the cut-off has been set to 6 months in order to have well balanced groups and a meaningful time interval. The evolution between D0 and D28 of the bacterial communities and diversity criteria was made with paired Wilcoxon ranked sum tests. All the *p*-values obtained from the tests were associated with their *q*-values (*p*-values with False Discovery Rate (FDR) correction^51^, the median and quartiles in both groups of interest. When the response variable was binary (e.g., Improved vs Stagnated/Worsened), in addition to p-values and q-values, the Area Under the ROC Curve (AUC) have been added in order to give an idea about the discriminatory power of the variable (e.g. the ability to distinguish Improved or Stagnated/Worsened according to the relative abundance of a bacteria). The ROC curve is a graphical representation of the couple sensitivity/specificity obtained at various thresholds. The area under this curve is often used to have an estimation of the diagnostic performance of a binary classifier. An AUC of 1 corresponds to a perfect separation of the groups while an AUC of 0.5 is equivalent to a random classifier.

Heatmaps were performed with the package “pheatmap”^52^, on standardised (centring and scaling) relative frequencies to visualise variations between groups even for low-abundant bacteria. The principal component analysis (PCA) was performed with the package “FactoMineR”^53^, on standardised data with a selection of the most discriminating bacteria for the groups according to the evolution of the wound. The goal of this approach was both to see if groups of patients can be observed on the individuals plot and to see how variables influence the position of patients suggesting a link between them. No missing data were observed for bacterial communities and only one missing data was observed for the clinical variables of interest (depth of wound was missing for one patient at D0, comparison was made on N = 12 vs 11 in this case).

## Supplementary Information

Supplementary Information 1.

Supplementary Information 2.

Supplementary Information 3.

Supplementary Information 4.

Supplementary Information 5.

Supplementary Information 6.

## Data Availability

The datasets generated and/or analysed during the current study are presented in Tables S1, S2 and S3 (summarising accession number of sequences).
