# Supplementary material for: Pressure ulcers microbiota dynamics and wound evolution
Source: Sci Rep. 2021 Sep 16;11:18506. doi: 10.1038/s41598-021-98073-x (PMC8445962; doi:10.1038/s41598-021-98073-x)
Supplement: Supplementary file 1 — Supplementary Information 1. [file 41598_2021_98073_MOESM1_ESM.docx]

**Additional files**

**Table S1:** Detailed table with clinical characteristics of the patients, the number of reads at D0/D28, the number of rDNA 16S reads assembled at 97% similarity at D0/D28, and the percentage of rDNA 16S reads assembled at 97% similarity at D0/D28.

**Table S2:** OTUs table corresponding to the repartition of relative abundance of OTU (%) for each patient and each time. Chao’s and Shannon’s Index are represented in the last lines of table.

**Table S3:** Accession numbers of sequences at D0 and D28 for 24 patients analysed in this study.

**Table S4:** Wound microbiota description in accordance to clinical data and characteristics of the wounds at enrolment (D0).

**Table S5:** Evolution of bacterial microbiota isolated from pressure ulcers over time.

**Table S6:** Wound microbiota description according to treatment (between D0 and D28) at D28.

**Figure S1:** Principal Coordinate Analysis (PCoA) based on Brain-Curtis distances for D0 and D28 biopsies samples. Samples are colored according to wound's evolution (Improved/Stagnated/Worsened) and day of visit (D0/D28).

**Figure S2:** Boxplot of relative frequencies for genera according to the evolution of wounds.

**Figure S3:** Evolution of *Proteus* and *Corynebacterium* over time (D0/D28).
