# Supplementary material for: Pressure ulcers microbiota dynamics and wound evolution
Source: Sci Rep. 2021 Sep 16;11:18506. doi: 10.1038/s41598-021-98073-x (PMC8445962; doi:10.1038/s41598-021-98073-x)
Supplement: Supplementary file 2 — Supplementary Information 2. [file 41598_2021_98073_MOESM2_ESM.pdf]

Id	Gender	Age	Body Mass Index (kg/m2)	Weight (D0)	Weight (D28)	Weight change (%)	Undernutrition Score	CRP (D0)	CRP (D28)	Wound stage (D0)	Wound stage (D28)	Wound localisation	Area of wound (D0)	Area of wound (D28)	Area of wound (% reduction)	Depth of wound (D0)	Depth of wound (D28)	Depth of wound (% reduction)	Duration of wound (D0)	Detachment (D0)	Detachment (D28)	Amount of exudate (D0)	Initial treatment	Antibiotherapy	Clinical evoluion of wound at D28	Nb of reads (D0)	Nb DNA 16S reads assembled at 97% similarity (D0)	Percentage of rDNA 16S reads assembled at 97% similarity (D0)	Nb of reads (D28)	Nb rDNA 16S reads assembled at 97% similarity (D28)	Percentage of rDNA 16Sreads assembled at 97% similarity (D28)
**C01P001**	Female	56	33.7	93	94	1,08	Moderate	16	20	4	4	Sacral	879,2	376,8	-57,14	20	10	-50,00	269	Yes	Yes	3	Yes	No	Improved	57812	48609	84,08%	59918	52413	87,47%
**C01P003**	Male	62	17.5	53	58	9,43	Severe	17	8	3	4	Ischial	392,5	451,375	15,00	10	15	50,00	32	Yes	Yes	2	Yes	No	Worsened	48660	39779	81,75%	50438	43412	86,07%
**C01P004**	Male	67	24.2	66	64	-3,03	None	22	26	3	3	Sacral	29437,5	31400	6,67	150	150	0,00	2083	No	No	2	No	No	Stagnated	46043	27255	59,19%	49731	41440	83,33%
**C01P005**	Female	76	24.8	58	57	-1,72	Moderate	26	23	3	2	Sacral	2041	706,5	-65,38	4	2	-50,00	927	No	No	4	No	Yes	Improved	56908	46974	82,54%	44315	37976	85,70%
**C01P007**	Male	52	24.5	75	75	0,00	None	9	22	3	3	Sacral	628	506,325	-19,38	3	5	66,67	54	No	No	2	Yes	No	Stagnated	12281	10194	83,01%	50161	43625	86,97%
**C01P008**	Female	83	27.2	74	74	0,00	None	3	4	3	3	Ischial	196,25	172,7	-12,00	15	22	46,67	183	No	No	3	No	No	Worsened	52602	44003	83,65%	52612	39553	75,18%
**C01P013**	Male	54	27.2	97	97	0,00	Moderate	67	14	3	3	Trochanteric	1511,125	989,1	-34,55	5	0	-100,00	445	No	No	3	No	No	Stagnated	50848	34212	67,28%	10444	6221	59,57%
**C01P014**	Male	31	37.9	116	116	0,00	None	6	7	4	4	Sacral	314	235,5	-25,00	30	40	33,33	1063	Yes	Yes	2	No	No	Worsened	48459	43633	90,04%	52081	46685	89,64%
**C01P015**	Female	56	17.7	53	54	1,89	Moderate	35	39	4	4	Ischial	414,48	102,05	-75,38	60	40	-33,33	190	Yes	Yes	3	Yes	No	Improved	49119	35246	71,76%	38032	32939	86,61%
**C01P016**	Female	68	30.1	82	82	0,00	Severe	7	4	3	3	Sacral	4906,25	2669	-45,60	27	10	-62,96	191	Yes	Yes	2	No	No	Improved	48850	27791	56,89%	45908	40311	87,81%
**C01P020**	Female	66	25.0	64	63	-1,56	Moderate	6	5	4	4	Sacral	294,375	176,625	-40,00	25	10	-60,00	207	Yes	Yes	2	Yes	No	Improved	56249	43614	77,54%	41122	31851	77,45%
**C01P029**	Male	49	31.7	111	111	0,00	Severe	11	1	3	3	Ischial	266,9	282,6	5,88	3	1	-66,67	195	No	No	2	No	No	Stagnated	56412	44962	79,70%	55054	44907	81,57%
**C01P030**	Female	89	30.4	74	73	-1,35	Severe	163	82	3	3	Sacral	2362,85	1413	-40,20	50	4	-92,00	154	Yes	Yes	4	Yes	No	Improved	49756	40045	80,48%	43760	37982	86,80%
**C01P031**	Male	65	29.4	89	93	4,49	None	13	13	3	3	Ischial	1884	1000,875	-46,88	0	0	0,00	4358	No	No	4	Yes	No	Improved	49562	41496	83,73%	45400	39445	86,88%
**C01P036**	Female	75	23.2	61	61	0,00	Severe	58	6	3	3	Sacral	3532,5	1632,8	-53,78	45	37	-17,78	52	No	Yes	3	Yes	Yes	Improved	59502	48046	80,75%	49585	42347	85,40%
**C01P037**	Male	66	29.8	83	82	-1,20	Severe	154	69	3	3	Ischial	381,51	440,385	15,43	45	44	-2,22	150	No	Yes	4	Yes	Yes	Stagnated	59577	43832	73,57%	51766	33367	64,46%
**C01P038**	Male	58	25.2	80	80	0,00	None	2	1	4	3	Ischial	133,45	157	17,65	18	20	11,11	50	Yes	Yes	3	Yes	No	Improved	49272	41624	84,48%	50670	43609	86,06%
**C01P040**	Male	47	21.9	75	64	-14,67	Severe	13	NA	4	4	Ischial	0,785	11,775	1400,00	2	10	400,00	20	Yes	Yes	3	No	No	Stagnated	56253	46699	83,02%	39894	33198	83,22%
**C01P041**	Male	61	25.4	75	75	0,00	None	11	10	3	3	Sacral	753,6	518,1	-31,25	2	9	350,00	794	Yes	No	2	No	No	Improved	45748	39028	85,31%	47247	40837	86,43%
**C01P043**	Female	67	18.7	45	NA	NA	Moderate	4	4	3	2	Sacral	106,76	23,55	-77,94	NA	4	NA	61	No	No	2	Yes	Yes	Improved	44482	26467	59,50%	41157	26464	64,30%
**C01P046**	Male	57	28.7	88	94	6,82	None	24	23	3	3	Ischial	2,355	180,55	7566,67	0	2	100,00	82	No	No	3	Yes	No	Improved	58967	48273	81,86%	42037	34394	81,82%
**C01P047**	Male	47	22.0	72	71	-1,39	Severe	75	21	4	4	Ischial	4239	5459,675	28,80	3	0	-100,00	126	Yes	Yes	4	No	No	Improved	50710	38121	75,17%	42402	24765	58,41%
**C01P048**	Male	61	27.4	83	86	3,61	Severe	62	24	3	3	Ischial	2755,35	8647,56	213,85	17	10	-41,18	195	Yes	No	4	No	Yes	Improved	47940	29052	60,60%	49170	31567	64,20%
**C01P049**	Male	64	25.1	87	88	1,15	None	22	28	4	3	Ischial	117,75	153,075	30,00	20	12	-40,00	637	Yes	Yes	3	No	No	Improved	48650	39390	80,97%	46095	36960	80,18%
