# Supplementary material for: Pressure ulcers microbiota dynamics and wound evolution
Source: Sci Rep. 2021 Sep 16;11:18506. doi: 10.1038/s41598-021-98073-x (PMC8445962; doi:10.1038/s41598-021-98073-x)
Supplement: Supplementary file 4 — Supplementary Information 4. [file 41598_2021_98073_MOESM4_ESM.pdf]

Accession	Sample Name	Isolate
SAMN20338432	C01P01_J0_R1	chronic pressure ulcer wounds
SAMN20338433	C01P01_J0_R2	chronic pressure ulcer wounds
SAMN20338434	C01P03_J0_R1	chronic pressure ulcer wounds
SAMN20338435	C01P03_J0_R2	chronic pressure ulcer wounds
SAMN20338436	C01P04_J0_R1	chronic pressure ulcer wounds
SAMN20338437	C01P04_J0_R2	chronic pressure ulcer wounds
SAMN20338438	C01P05_J0_R1	chronic pressure ulcer wounds
SAMN20338439	C01P05_J0_R2	chronic pressure ulcer wounds
SAMN20338440	C01P07_J0_R1	chronic pressure ulcer wounds
SAMN20338441	C01P07_J0_R2	chronic pressure ulcer wounds
SAMN20338442	C01P08_J0_R1	chronic pressure ulcer wounds
SAMN20338443	C01P08_J0_R2	chronic pressure ulcer wounds
SAMN20338444	C01P13_J0_R1	chronic pressure ulcer wounds
SAMN20338445	C01P13_J0_R2	chronic pressure ulcer wounds
SAMN20338446	C01P14_J0_R1	chronic pressure ulcer wounds
SAMN20338447	C01P14_J0_R2	chronic pressure ulcer wounds
SAMN20338448	C01P15_J0_R1	chronic pressure ulcer wounds
SAMN20338449	C01P15_J0_R2	chronic pressure ulcer wounds
SAMN20338450	C01P16_J0_R1	chronic pressure ulcer wounds
SAMN20338451	C01P16_J0_R2	chronic pressure ulcer wounds
SAMN20338452	C01P20_J0_R1	chronic pressure ulcer wounds
SAMN20338453	C01P20_J0_R2	chronic pressure ulcer wounds
SAMN20338454	C01P29_J0_R1	chronic pressure ulcer wounds
SAMN20338455	C01P29_J0_R2	chronic pressure ulcer wounds
SAMN20338456	C01P30_J0_R1	chronic pressure ulcer wounds
SAMN20338457	C01P30_J0_R2	chronic pressure ulcer wounds
SAMN20338458	C01P31_J0_R1	chronic pressure ulcer wounds
SAMN20338459	C01P31_J0_R2	chronic pressure ulcer wounds
SAMN20338460	C01P36_J0_R1	chronic pressure ulcer wounds
SAMN20338461	C01P36_J0_R2	chronic pressure ulcer wounds
SAMN20338462	C01P37_J0_R1	chronic pressure ulcer wounds
SAMN20338463	C01P37_J0_R2	chronic pressure ulcer wounds
SAMN20338464	C01P38_J0_R1	chronic pressure ulcer wounds
SAMN20338465	C01P38_J0_R2	chronic pressure ulcer wounds
SAMN20338466	C01P40_J0_R1	chronic pressure ulcer wounds
SAMN20338467	C01P40_J0_R2	chronic pressure ulcer wounds
SAMN20338468	C01P41_J0_R1	chronic pressure ulcer wounds
SAMN20338469	C01P41_J0_R2	chronic pressure ulcer wounds
SAMN20338470	C01P43_J0_R1	chronic pressure ulcer wounds
SAMN20338471	C01P43_J0_R2	chronic pressure ulcer wounds
SAMN20338472	C01P46_J0_R1	chronic pressure ulcer wounds
SAMN20338473	C01P46_J0_R2	chronic pressure ulcer wounds
SAMN20338474	C01P47_J0_R1	chronic pressure ulcer wounds
SAMN20338475	C01P47_J0_R2	chronic pressure ulcer wounds
SAMN20338476	C01P48_J0_R1	chronic pressure ulcer wounds
SAMN20338477	C01P48_J0_R2	chronic pressure ulcer wounds
SAMN20338478	C01P49_J0_R1	chronic pressure ulcer wounds

SAMN20338479	C01P49_J0_R2	chronic pressure ulcer wounds
SAMN20338480	C01P01_J28_R1	chronic pressure ulcer wounds
SAMN20338481	C01P01_J28_R2	chronic pressure ulcer wounds
SAMN20338482	C01P03_J28_R1	chronic pressure ulcer wounds
SAMN20338483	C01P03_J28_R2	chronic pressure ulcer wounds
SAMN20338484	C01P04_J28_R1	chronic pressure ulcer wounds
SAMN20338485	C01P04_J28_R2	chronic pressure ulcer wounds
SAMN20338486	C01P05_J28_R1	chronic pressure ulcer wounds
SAMN20338487	C01P05_J28_R2	chronic pressure ulcer wounds
SAMN20338488	C01P07_J28_R1	chronic pressure ulcer wounds
SAMN20338489	C01P07_J28_R2	chronic pressure ulcer wounds
SAMN20338490	C01P08_J28_R1	chronic pressure ulcer wounds
SAMN20338491	C01P08_J28_R2	chronic pressure ulcer wounds
SAMN20338492	C01P13_J28_R1	chronic pressure ulcer wounds
SAMN20338493	C01P13_J28_R2	chronic pressure ulcer wounds
SAMN20338494	C01P14_J28_R1	chronic pressure ulcer wounds
SAMN20338495	C01P14_J28_R2	chronic pressure ulcer wounds
SAMN20338496	C01P15_J28_R1	chronic pressure ulcer wounds
SAMN20338497	C01P15_J28_R2	chronic pressure ulcer wounds
SAMN20338498	C01P16_J28_R1	chronic pressure ulcer wounds
SAMN20338499	C01P16_J28_R2	chronic pressure ulcer wounds
SAMN20338500	C01P20_J28_R1	chronic pressure ulcer wounds
SAMN20338501	C01P20_J28_R2	chronic pressure ulcer wounds
SAMN20338502	C01P29_J28_R1	chronic pressure ulcer wounds
SAMN20338503	C01P29_J28_R2	chronic pressure ulcer wounds
SAMN20338504	C01P30_J28_R1	chronic pressure ulcer wounds
SAMN20338505	C01P30_J28_R2	chronic pressure ulcer wounds
SAMN20338506	C01P31_J28_R1	chronic pressure ulcer wounds
SAMN20338507	C01P31_J28_R2	chronic pressure ulcer wounds
SAMN20338508	C01P36_J28_R1	chronic pressure ulcer wounds
SAMN20338509	C01P36_J28_R2	chronic pressure ulcer wounds
SAMN20338510	C01P37_J28_R1	chronic pressure ulcer wounds
SAMN20338511	C01P37_J28_R2	chronic pressure ulcer wounds
SAMN20338512	C01P38_J28_R1	chronic pressure ulcer wounds
SAMN20338513	C01P38_J28_R2	chronic pressure ulcer wounds
SAMN20338514	C01P40_J28_R1	chronic pressure ulcer wounds
SAMN20338515	C01P40_J28_R2	chronic pressure ulcer wounds
SAMN20338516	C01P41_J28_R1	chronic pressure ulcer wounds
SAMN20338517	C01P41_J28_R2	chronic pressure ulcer wounds
SAMN20338518	C01P43_J28_R1	chronic pressure ulcer wounds
SAMN20338519	C01P43_J28_R2	chronic pressure ulcer wounds
SAMN20338520	C01P46_J28_R1	chronic pressure ulcer wounds
SAMN20338521	C01P46_J28_R2	chronic pressure ulcer wounds
SAMN20338522	C01P47_J28_R1	chronic pressure ulcer wounds
SAMN20338523	C01P47_J28_R2	chronic pressure ulcer wounds
SAMN20338524	C01P48_J28_R1	chronic pressure ulcer wounds
SAMN20338525	C01P48_J28_R2	chronic pressure ulcer wounds
SAMN20338526	C01P49_J28_R1	chronic pressure ulcer wounds

SAMN20338527	C01P49_J28_R2	chronic pressure ulcer wounds
