# Supplementary material for: Pressure ulcers microbiota dynamics and wound evolution
Source: Sci Rep. 2021 Sep 16;11:18506. doi: 10.1038/s41598-021-98073-x (PMC8445962; doi:10.1038/s41598-021-98073-x)
Supplement: Supplementary file 5 — Supplementary Information 5. [file 41598_2021_98073_MOESM5_ESM.docx]

Table S4: Wound microbiota description according to clinical data and wound characteristics at D0.

|  | |  | | | |  | | **p** | | **q, AUC** |
| --- | --- | --- | --- | --- | --- | --- | --- | --- | --- | --- |
| **Duration of PU (months) D0** |  | | **≤6**  (N=10) | | **>6**  (N=14) | |  | |  | |
|  |  | |  | |  | |  |  |  |  |
| -Genus^a^ |  | | NS | | NS | |  | | q=0.84, AUC=0.58 | |
| -Chao’s index |  | | 75 (50, 186) | | 75.5 (46.5, 132.5) | | p=0.5 | |  | |
| **Depth of wound (D0) (mm)**  -Genus^a^  *Dietza*  *Oscillospira*  *Paracoccus*  *Novosphingbomium*  *Delftia*  *Pelomonas*  -Chao’s index |  | | **>15**  (N=12)  0 (0, 0.015)  0 (0, 0.01)  0 (0, 0.032)  0.025 (0, 0.07)  0.24 (0.07, 0.5)  0.03 (0, 0.075)  96 (69, 175.2) | | **≤15**  (N= 12)  0 (0, 0)  0 (0, 0)  0 (0, 0)  0 (0, 0)  0.05 (0, 0.06)  0 (0, 0.005)  40 (33, 79) | | p=0.045,  p=0.045  p=0.045  **p=0.003**  **p=0.016**  **p=0.0049**  **p=0.03** | | q=0.54, AUC=0.667  q=0.54, AUC=0.667  q=0.54, AUC=0.667  q=0.424, AUC=0.833  q=0.537, AUC=0.8 q=0.537, AUC=0.723  **q=0.047, AUC=0.77** | |
| **Area of wound (mm) (D0)**  -Genus^a^  *Brevibacterium*  -Chao’s index |  | | **>600**  (N=12)  0 (0, 0)  69.5 (38.2, 193.2) | | **≤600**  (N=12)  0 (0, 0.022)  79 (63.5, 121.5) | | **p=0.037**  p=0.98 | | q=0.6, AUC=0.667  q=1, AUC=0.51 | |
| **Wound stage (D0)**  -Genus^a^  *Dermabacter*  *Peptococcus*  Other *Peptostreptococcaceae*  *Peptostreptococaceae*  -Chao’s index |  | | III  (N=16)  0.035 (0, 0.51)  0 (0, 0)  0 (0, 0)  0 (0, 0)  75.5 (39.7, 162.7) | | IV  (N= 8)  0 (0, 0.005)  0 (0, 0.21)  0 (0, 0.021)  0 (0, 0.005)  75 (52, 125.7) | | **p=0.042**  **p=0.01**  **p=0.01**  **p=0.048**  p=0.85 | | q=0.69, AUC=0.75  q=0.69, AUC=0.69  q=0.69, AUC=0.69  q=0.69, AUC=0.62  q=0.85, AUC=0.53 | |
| **Malnutrition (D0)**  -Genus^a^  *Brevibacterium*  Other *Gemellaceae*  *Finegoldia*  -Chao’s index |  | | No  (N=9)  0 (0, 0.3)  0 (0, 0.09)  1.53 (1.04, 14.31)  67 (36, 78) | Moderate or severe  (N =15)  0 (0,0)  0 (0,0)  0.2 (0.005, 2.75)  112 (57.5, 167.5) | | | **p=0.007**  **p=0.029**  **p=0.025**  p=0.15 | | q=0.73, AUC=0.722  q=0.73, AUC=0.7  q=0,73, AUC= 0.78  q=0.46, AUC=0.68 | |

^a^genus with significantly different relative abundance according to wound characteristics or clinical data, bold: p<0.05. P values come from Wilcoxon-Mann-Whitney tests. q-values are corrected p-values with FDR. AUC is the area under a ROC curve.

Tables S5: Evolution of bacterial microbiota isolated from wounds over time.

| **Variable** | | **D0^a^** | **D28^a^** | **p-value^b^** | **q-value** |
| --- | --- | --- | --- | --- | --- |
| **Diversity (Chao’s index)** | Total population | 75.5 (46.7, 156.5) | 40 (23, 107) | 0.1 | 0.1 |
|  | With ATB (n=5) | 150 (73, 153) | 138 (40, 146) | 0.4375 |  |
|  | Improved (n=15) | 78.0 (57.5, 158.5) | 40 (23, 113) | 0.1728 |  |
|  | Stagnated/Worsened (n=9) | 67 (40, 153) | 41.5 (27.0, 94.5) | 0.4406 |  |
| **Phylum: *Firmicutes*** | *Staphylococcus* | 1.24 (0.048, 29.0) | 1.05 (0.035, 69.5) | 0.578 | 1 |
|  | *Streptococcus* | 0.22 (0.045, 11.6) | 0.30 (0.0075, 10.75) | 0.185 | 1 |
|  | *Enterococcus* | 0.175 (0, 0.73) | 0.025 (0, 7.8) | 0.82 | 1 |
|  | *Anaerococcus* | 0.34 (0.018, 3.3) | 0.085 (0.075, 2) | **0.04** | 1 |
|  | *Peptoniphilus* | 0.65 (0.01, 3,15) | 0.06 (0.007, 1.18) | 0.24 | 1 |
| **Phylum: *Proteobacteria*** | *Sphingomonas* | 0.06 (0.01, 0.38) | 0.01 (0, 0.11) | **0.02** | 1 |
|  | *Delftia* | 0.08 (0.017, 0.23) | 0.025 (0.01, 0.06) | **0.05** | 1 |
| **Phenotype** | Aero-anaerobic | 61.72 (11.7, 91) | 89.58 (26, 98.6) | **0.02** | 0.07 |
|  | Anaerobic | 4.18 (0.27, 42.15) | 0.72 (0.06, 19.8) | 0.25 | 0.25 |
|  | Aerobic | 1.13 (0.13, 7.36) | 0.17 (0.04, 4.13) | 0.23 | 0.25 |
|  | Cocci | 47.5 (27.72, 85.24) | 89.96 (35.7, 98.6) | **0.019** | **0.07** |
|  | Rods | 17.4 (1.9, 46.0) | 6.37 (0.24, 26.51) | 0.053 | 0.07 |

^a^Median (Q1,Q3)

^b^P values were calculated with paired Wilcoxon rank sum tests. q-values are corrected p-values with FDR.

Table S6: wound microbiota description according to treatment (between D0 and D28) at D28.

|  | |  | | |  | | **p** | | **q, AUC** |
| --- | --- | --- | --- | --- | --- | --- | --- | --- | --- |
| **Antibiotherapy (D28)**  -Genus  *Propionibacterrium*  *Meiothermus*  -Chao’s index |  | | **ATB**  (N=5)  0 (0, 0.06)  0 (0, 0.07)  138 (40, 146) | **No**  (N= 19)  0 (0, 0)  0 (0, 0)  36 (22, 0.72) | | **p=0.035**  **p=0.006**  p=0.28 | | q=0.75, AUC=0.68  q=0.75, AUC=0.70  **q=0.84, AUC=0.66** | |
| **Dressing (D28)**  -Genus  *Bifidobacterium*  -Chao’s index |  | | **Alginate**  (N=11)  0 (0, 0.015)  45.5 (35.2, 81.5) | **Hydro^b^**  (N=10)  0 (0, 0.0)  35 (22, 111) | | **p=0.045**  p=0.326 | | q=0.5, AUC=0.68  q=0.49, AUC=0.63 | |

^a^ATB: antibiotics

^b^Hydro: hydrocellular, hydrofiber, hydrocolloid dressings

^c^Other: other than alginate, hydrocellular, hydrofiber, hydrocolloid dressings
