# Supplementary material for: Pressure ulcers microbiota dynamics and wound evolution
Source: Sci Rep. 2021 Sep 16;11:18506. doi: 10.1038/s41598-021-98073-x (PMC8445962; doi:10.1038/s41598-021-98073-x)
Supplement: Supplementary file 6 — Supplementary Information 6. [file 41598_2021_98073_MOESM6_ESM.docx]

Fig S1

Fig S2

Fig S3
